# Editorial: Female Infertility: Genetics of Reproductive Ageing, Menopause and Primary Ovarian Insufficiency

**DOI:** 10.3389/fgene.2022.839758

**Published:** 2022-03-09

**Authors:** Mara Marongiu, Laura Crisponi, Manuela Uda, Emanuele Pelosi

**Affiliations:** ^1^ Institute of Genetic and Biomedical Research, National Research Council (CNR), Monserrato (Cagliari), Italy; ^2^ The University of Queensland, Brisbane, QLD, Australia

**Keywords:** female infertility, age-related decline in fertility, reproductive aging, hypothalamic-pituitary-ovarian axis, primary ovarian insufficiency (POI), ovarian biology

## Introduction

Worldwide, infertility affects between 8 and 12% of reproductive-aged couples (Vander Borght and Wyns, 2018). Female infertility represents a growing health problem, especially in industrialized countries, where the ongoing trends of delaying pregnancy beyond 35 years of age significantly reduce fertility rates.

The age-related decline in fertility is characterized by a gradual decrease in the number of primordial follicles. Menopause ensues around 51 years of age and is exemplified by the cessation of ovarian functions. However, fertility begins to decline around 30 years of age in women. Therefore, understanding the molecular mechanisms underlying the age-related decline in fertility and ovarian function and associated conditions, including primary ovarian insufficiency (POI), reproductive aging, and menopause, is critical to find ways to preserve fertility and manage comorbidities related to the premature onset of estradiol deficiency.

With this research topic (RT), we aimed to tackle the issue of female infertility from different perspectives related to age, endocrine imbalances, and genetic disorders, particularly the early decline in ovarian function such as in POI.

### Articles Contributions

This RT includes 13 papers: five original research articles, one brief research report, five reviews, one case report, and one clinical trial.

Early decline in ovarian function occurs in around 10% of women and is described by a markedly reduced follicular reserve and ovarian response. POI, previously referred to as “premature ovarian failure,” is characterized by impairment of ovarian function before 40 years and affects approximately 1% of women under 40 and 0.1% of women under 30. POI is a heterogeneous condition due to genetic and non-genetic factors, such as autoimmunity (e.g., oophoritis), environmental toxins, and chemicals. Attrition in the number of residual ovarian follicles and ensuing deficiency in ovarian sex hormones are hallmarks of POI, thus rendering a woman subfertile and estrogen-deficient several years (even decades) before the average age of menopause. Clinical presentations of POI can also be due to FSH/LH receptor mutations leading to “resistant ovaries” retaining normal follicles in otherwise normal ovaries.

In contrast, POI cases with no detectable FSH/LH receptor mutations show a deficit in ovarian follicles. These vary from complete failure of germ-cell development, resulting in primary amenorrhea, to an accelerated decline in germ cell numbers, leading to the cessation of ovarian function before age 40 (Crisponi et al., 2001). Several genetic factors have been reported in such cases, including *FOXL2, STAG3, FOXO3a*, and X-linked genes, such as *FMR1* and *BMP15*. However, POI remains poorly understood to date, with 90% of cases still with unknown causes (ESHRE Guideline, 2016).

Piedade et al. provided an excellent review of genetic disorders associated with POI and evaluated the clinical phenotypes and molecular mechanisms of POI. The authors also addressed the quality of life, emotional health, and quality of care of women with Overt POI. Importantly, they suggested methods to optimize fertility in these women. They also described a case report of a successful pregnancy and birth achieved by follicle monitoring in a woman with Overt POI. The method is based on the hypothesis that employing the NIH P-HRT regimen to suppress luteinizing hormone (LH) levels could prevent follicle luteinization, restore follicle function and ovulation, and increase the chance of pregnancy in women with Overt POI.

Jiao et al. characterized ovarian response indicators in women with POI. The authors focused on AMH and AFC and concluded that changes in their levels could predict pre-POI and facilitate early diagnosis.

Rossetti et al. used target next-generation sequencing with a panel of 295 known and novel candidates to study the genetics of 64 POI patients. They identified 34 novels and 9 already known variants, suggesting an oligogenic nature of POI.

Louwers and Visser described genetic determinants in common between age at menopause, early menopause, POI, and other traits, highlighting that a critical analysis of genetic variants and methods used is essential and that multidisciplinary research teams are necessary for proper study design and result interpretation.

In certain rare and specific cases, defects in biological systems important in maintaining DNA integrity appear to be involved in the age-related decline of ovarian function.

Homer reviewed data from the Senataxin knockout mouse model (*Setx* ^*−/−*^)*,* pointing to the importance of SETX in delaying the age-related decline in ovarian function and discussing the implications for understanding this phenomenon in humans. SETX is an RNA/DNA helicase involved in repairing oxidative stress-induced DNA damage. It is well known for its role in preventing neurodegenerative disease, and it has recently been found involved in male fertility by maintaining genomic integrity during spermatogenesis. Although dispensable for oogenesis, mouse SETX is critical for protecting oocyte DNA integrity and exhibits a unique role in slowing the age-related decline in ovarian function.

The age-related decline in fertility is not necessarily of ovarian origin. For instance, there is a distinct possibility of the central nervous system changes playing a role.

Nicola et al. arose an additional link between aging and fertility involving circadian clocks. The capacity of circadian rhythm regulator genes seems reduced during the senescence period, and previous studies showed the importance of the suprachiasmatic nucleus (SCN) in regulating the circadian system in aged organisms (Satinoff et al., 1993; Cai et al., 1997; Li and Satinoff, 1998). The involvement of the SCN and the activity of vasopressinergic neurons in maintaining the rhythmicity of the female reproductive system depends on the mRNA transcription-translation feedback loops. Therefore, circadian clock function is involved in the events determining age-related decline in fertility and ovarian function like most physiological processes. Nicola et al. demonstrated that the feedback loops of clock genes on the hypothalamus-pituitary-gonadal axis (HPG) modulate cyclicity in female rodents and that the desynchronization between the central and peripheral circadian clocks contributes to the irregularity of reproductive events.

Guo et al. analyzed the ubiquitously expressed miR-29 family in mice. The authors report that female miR-29a/b1 knockout mice exhibit severe fertility problems, possibly due to disrupted secretion of the luteinizing hormone leading to ovulation failure and subfertility.

Some of the age-related fertility declines are related to embryonic events.

Zhang et al., Gu et al., and Sun et al. analyzed different aspects of infertility related to preimplantation embryo lethality (PEL), pregnancy loss (<11 gestational weeks), and spontaneous abortions (<20 weeks). Zhang et al. described a novel biallelic transducin-like enhancer of the split 6 (TLE6) variant in a cohort of patients with PEL. TLE6 is a transcriptional co-repressors component of the subcortical maternal complex (SCMC). Recent evidence from ART and embryo research suggests that PEL may be a rare cause of primary female infertility (Yatsenko and Rajkovic, 2019).

Gu et al. conducted a retrospective study on 1,102 women who experienced singleton pregnancy loss and underwent chromosomal microarray analysis (CMA). Their study demonstrated that pregnancy loss in women over 35 is associated with a higher chromosomal aneuploidy rate and increased autosomal trisomy. Chromosome trisomy mainly results from un-separated chromosomes in oogenesis, related to advanced maternal age. The strengths of this study include its population-based setting and relatively large sample size.

This RT also includes manuscripts on PCOS, the most common endocrine-metabolic disorder causing infertility due to anovulation. Worldwide its prevalence ranges from 4 to 21% depending on the diagnostic criteria (Lizneva et al., 2016).

Sun et al. aimed to assess the impact of relevant risk factors on spontaneous abortion in patients with polycystic ovary syndrome (PCOS). The authors examined the correlation of spontaneous abortions in patients undergoing assisted reproductive treatment (ART) with risk factors including body mass index (BMI), age, hyperandrogenism, insulin resistance (IR), and chromosome aberrations. Their analysis concluded that high BMI and insulin resistance are two risk factors for increased spontaneous abortion in PCOS patients undergoing ART.

In an independent study, Qorbani et al. analyzed the effects of oligopin supplementation on hormonal and metabolic profiles in PCOS by a randomized controlled trial (RCT). Oligopin is a plant extract with strong antioxidant and anti-inflammatory activity (Sedighiyan et al., 2018). However, according to the authors, oligopin supplementation does not benefit women’s hormonal and metabolic parameters.

The involvement of FOXO1 in PCOS has been reviewed by Xu et al. FOXO1, a member of the forkhead transcription factor family (FoxO), plays a vital role during glycolipid metabolism, IR, and oxidative stress. Since PCOS has been associated with IR and low-grade inflammatory response, the authors support the need to clarify the role of FOXO1.

Hong Zhang et al. detailed the function of another FoxO, FOXO3, in the physiological regulation of ovarian follicular development. Their contribution provides an important reference for further studies of ovarian biology, including valuable insights for the modulation of the age-related decline in ovarian function, reproductive lifespan, and ovarian disease.

## Conclusion

The results presented in this RT summarize some of the latest findings in infertility research and the regulation of ovarian biology, underlying the role of genes affecting the age-related decline in fertility (i.e., the well-known FOXO factors and SETX) and embryonic lethality (i.e., TLE6), and highlighting emerging mechanisms including the regulation of ovarian functions by circadian rhythms and miRNAs.

These reports highlight the critical role of aging in female infertility through several mechanisms affecting ovarian function, including the HPG axis, the role of hormones, integrity of meiotic and mitotic divisions, thus affecting follicle dynamics (changes in follicle numbers due to processes including oocyte awakening, primordial follicle activation, follicular growth and maturation), preimplantation embryo lethality, pregnancy loss, and aneuploidy of conception products.
